# Single Ethanol Withdrawal Regulates Extrasynaptic δ-GABA_A_ Receptors Via PKCδ Activation

**DOI:** 10.3389/fnmol.2018.00141

**Published:** 2018-04-26

**Authors:** Juan Chen, Yang He, Yan Wu, Hang Zhou, Li-Da Su, Wei-Nan Li, Richard W. Olsen, Jing Liang, Yu-Dong Zhou, Yi Shen

**Affiliations:** ^1^Department of Neurobiology, Institute of Neuroscience, Key Laboratory of Medical Neurobiology of the Ministry of Health of China, Zhejiang University School of Medicine, Hangzhou, China; ^2^Neuroscience Care Unit, Second Affiliated Hospital of Zhejiang University School of Medicine, Hangzhou, China; ^3^Department of Molecular & Medical Pharmacology, David Geffen School of Medicine, University of California, Los Angeles, Los Angeles, CA, United States; ^4^Titus Family Department of Clinical Pharmacy, USC School of Pharmacy, University of Southern California, Los Angeles, CA, United States

**Keywords:** ethanol withdrawal, extrasynaptic δ-GABA_A_R, tonic current, PKCδ, hippocampal neurons

## Abstract

Alcohol (ethanol, EtOH) is one of the most widely abused drugs with profound effects on brain function and behavior. GABA_A_ receptors (GABA_A_Rs) are one of the major targets for EtOH in the brain. Temporary plastic changes in GABA_A_Rs after withdrawal from a single EtOH exposure occur both *in vivo* and *in vitro*, which may be the basis for chronic EtOH addiction, tolerance and withdrawal symptoms. Extrasynaptic δ-GABA_A_R endocytosis is implicated in EtOH-induced GABA_A_R plasticity, but the mechanisms by which the relative abundance and localization of specific GABA_A_Rs are altered by EtOH exposure and withdrawal remain unclear. In this study, we investigated the mechanisms underlying rapid regulation of extrasynaptic δ-GABA_A_R by a single EtOH withdrawal in cultured rat hippocampal neurons. Thirty-minutes EtOH (60 mM) exposure increased extrasynaptic tonic current (*I*_tonic_) amplitude without affecting synaptic GABA_A_R function in neurons. In contrast, at 30 min after withdrawal, *I*_tonic_ amplitude and responsiveness to acute EtOH were both reduced. Similar results occurred in neurons with okadaic acid (OA) or phorbol 12,13-dibutyrate (PDBu) exposure. Protein kinase C (PKC) inhibition prevented the reduction of *I*_tonic_ amplitude and the tolerance to acute EtOH, as well as the reduction of GABA_A_R-δ subunit abundance induced by a single EtOH withdrawal. Moreover, EtOH withdrawal selectively increased PKCδ level, whereas PKCδ inhibition specifically rescued the EtOH-induced alterations in GABA_A_R-δ subunit level and δ-GABA_A_R function. Together, we provided strong evidence for the important roles of PKCδ in the rapid regulation of extrasynaptic δ-GABA_A_R induced by a single EtOH withdrawal.

## Introduction

Alcohol (ethanol, EtOH) consumption has profound effects on brain function and behavior (Olsen et al., 2007; Abrahao et al., 2017). Continued excessive EtOH consumption can lead to the development of dependence that is associated with withdrawal symptoms when alcohol consumption is ceased or substantially reduced. EtOH acts on GABA_A_ receptors (GABA_A_Rs) as a major pharmacological target in the brain (Rudolph et al., 2001; Olsen et al., 2007; Olsen and Sieghart, 2008). GABA_A_Rs are ligand-gated chloride channels assembled into heteropentamers, which mediate the majority of inhibitory neurotransmission in the brain (Mehta and Ticku, 1999; Rudolph et al., 2001). GABA_A_R function can be allosterically enhanced by EtOH (Wallner et al., 2003; Olsen et al., 2007), and chronic activation of GABA_A_Rs by EtOH produces plastic changes that may contribute to EtOH tolerance, dependence and withdrawal symptoms (Liang et al., 2006; Kumar et al., 2009). GABA_A_Rs composed of α1–3 and γ2 subunits largely localize at synaptic sites and participate in the phasic (synaptic) inhibitory control of neuronal activity, whereas α4–6 and δ subunits are predominantly expressed extrasynaptically and mediate tonic (extrasynaptic) inhibition (Farrant and Nusser, 2005; Glykys et al., 2008; Brickley and Mody, 2012). Different GABA_A_R subunits have distinct localization, physiological and pharmacological properties, which account for variable sensitivity to GABA_A_R modulators and subsequent behavioral consequences (Puia et al., 1991; Whiting et al., 1999; Olsen and Sieghart, 2008).

Previous studies have elucidated that synaptic and extrasynaptic GABA_A_R subtypes are differentially modulated by EtOH (Sundstrom-Poromaa et al., 2002; Wallner et al., 2003; Wei et al., 2004). Extrasynaptic δ subunit-containing GABA_A_Rs (δ-GABA_A_Rs) are abundantly expressed in the hippocampus (Sperk et al., 1997; Peng et al., 2002) with unique properties such as high affinity for GABA, slow desensitization kinetics, benzodiazepine insensitivity and high sensitivity for EtOH (Sundstrom-Poromaa et al., 2002; Wallner et al., 2003; Wei et al., 2003, 2004; Mody and Pearce, 2004; Hanchar et al., 2005; Liang et al., 2007). GABA_A_R-δ subunits are important targets for EtOH (Olsen and Liang, 2017). δ deficient (δ^−/−^) mice show defects in their behavioral responses to EtOH, with reduced EtOH consumption, attenuated withdrawal from chronic EtOH exposure, and reduced anticonvulsant effects of EtOH (Mihalek et al., 2001); Chronic intermittent EtOH (CIE) treatment and withdrawal in rats results in decreased EtOH-enhanced δ-GABA_A_R-mediated tonic inhibitory current (*I*_tonic_) correlated to down-regulated δ subunit (Cagetti et al., 2003; Liang et al., 2004, 2006). Furthermore, a single intoxicating dose of EtOH administered by gavage is able to induce many of the same, but transient changes in behavior, GABA_A_R subunit composition, and hippocampal neuron pharmacology seen in CIE (Liang et al., 2007; Olsen and Spigelman, 2012; Olsen and Liang, 2017). This indicates that the plasticity induced by a single EtOH is likely to be helpful in figuring out what plasticity induced by chronic and/or repeated EtOH administration can be dependence-inducing.

The rapid (within 1-h withdrawal) down-regulation of δ-GABA_A_R function and cell-surface subunit level by a single EtOH primarily results from increased δ-GABA_A_R endocytosis rather than decreased surface insertion (Shen et al., 2011; Gonzalez et al., 2012). Phosphorylation regulation of GABA_A_Rs has been suggested in EtOH-mediated GABA_A_R plasticity (Kumar et al., 2009; Nakamura et al., 2015) for both synaptic γ2-GABA_A_Rs (Qi et al., 2007) and extrasynaptic δ-GABA_A_Rs (Choi et al., 2008). As a critical regulator of protein phosphorylation, protein kinase C (PKC) modulates the surface expression of both recombinant and native GABA_A_Rs in cultured neurons (Chapell et al., 1998) by endocytosis into clathrin-coated vesicles (CCVs; Kittler et al., 2000). Moreover, PKC plays important roles in facilitating EtOH regulation of GABA_A_Rs (Kumar et al., 2010, 2012; Werner et al., 2011), and in regulation of EtOH-mediated behavioral effects (Harris et al., 1995; Hodge et al., 1999; Choi et al., 2008). Previous studies in transgenic or knockout manipulation of PKC isoforms demonstrate that PKC isoforms may participate in EtOH-mediated GABA_A_R plasticity and behavioral effects: PKCε mutant mice are more sensitive to short-term EtOH exposure, whereas the deletion of PKCε attenuates EtOH withdrawal-associated seizures in mice (Olive et al., 2000); PKCγ knockout mice are resistant to the short-term intoxicating effects of EtOH and fail to develop EtOH tolerance (Harris et al., 1995; Bowers et al., 1999; Bowers and Wehner, 2001); PKCδ knockout mice have shown diminished acute responses to EtOH (Choi et al., 2008). However, whether PKC isoforms modulate the effects of a single EtOH withdrawal on δ-GABA_A_Rs remains to be investigated.

Here we show that PKC inhibition prevents the decreases in *I*_tonic_ amplitude and responsiveness to acute EtOH as well as the reduction in extrasynaptic δ subunit abundance induced by a single EtOH withdrawal. Moreover, EtOH withdrawal selectively increases PKCδ level in neurons, whereas PKCδ inhibition specifically blocked the EtOH withdrawal-induced alterations in protein level of δ subunits and δ-GABA_A_R function. Together, our findings highlight the importance of PKCδ in a single EtOH withdrawal-regulation of extrasynaptic δ-GABA_A_Rs.

## Materials and Methods

All procedures were carried out in accordance with the recommendations of National Institutes of Health Guidelines for the Care and Use of Laboratory Animals and were approved by the Animal Advisory Committee at Zhejiang University. For all materials involving biohazards, biological select agents, toxins and restricted materials or reagents used in the study, the standard bio-safety procedures were approved by the Division of Laboratory and Equipment Management at Zhejiang University.

### Primary Hippocampal Neuron Culture

The protocol was approved by the Animal Advisory Committee at Zhejiang University. Hippocampal neurons from embryonic 18 (E18) Sprague-Dawley rats were prepared as described previously (Shen et al., 2011). Briefly, embryos were removed from pregnant rats anesthetized with isoflurane and euthanized by decapitation. Hippocampi were dissected and placed in Ca^2+^- and Mg^2+^-free N-2-hydroxy-ethylpiperazine-N-2-ethanesulfonic acid (HEPES)-buffered Hank’s balanced salt solution (HBSS; pH 7.45), followed by a digestion with 0.25% w/v trypsin. After trituration through a Pasteur pipette, neurons were centrifuged (1000 *g* for 5 min) and resuspended in Neurobasal medium containing 2% B27 serum-free supplement, 1% v/v penicillin/streptomycin (P/S), 0.5 mM glutamine, and 10 μM glutamate (Sigma-Aldrich). Dissociated cells were then plated at a density of 0.03 × 10^6^ cells/cm^2^ onto 12-mm-diameter round coverslips in 24-well plates (Corning Costar^°ledR^, for recordings) and glass bottom confocal dishes (NEST Biotechnology, for live-cell imaging), as well as at a density of 0.05 × 10^6^ cells/cm^2^ in 150-mm-diameter dishes (for Western blots) pre-coated with poly-D-lysine (PDL, 50 μg/ml; Sigma-Aldrich). Cultures were kept at 37°C in a 5% v/v CO_2_ humidified incubator. Thereafter, one third to half of the medium was replaced twice a week with Neurobasal culture medium containing 2% B27 supplement, and 0.5 mM glutamine. All reagents were purchased from Thermo Fisher Scientific unless otherwise specified.

### EtOH and Drug Treatment

Cultured neurons at 13-14 days *in vitro* (DIV13-14; DIV9–10 for transfected-neurons) were used for EtOH and drug treatments. Before experiments, half of the culture medium was replaced with Neurobasal culture medium containing 120 mM EtOH (final concentration was 60 mM) for 30 min, and then the entire medium was replaced by half fresh Neurobasal culture medium plus half original medium. Control neurons were treated with corresponding vehicle using the same procedure as EtOH exposure and withdrawal. The test compounds including okadaic acid (OA, a phosphatase blocker, 100 nM); phorbol 12,13-dibutyrate (PDBu, a PKC activator, 100 nM) and forskolin (a PKA activator, 10 μM) were added in the culture medium for 30 min immediately before experiments, whereas the test compounds including chelerythrine chloride (Che, a PKC inhibitor, 10 μM), H-89 dihydrochloride hydrate (H89, a PKA inhibitor, 20 μM), and a selective PKCδ inhibitor Rottlerin (Enzo Life Science, 10 μM), and a selective PKCε inhibitor peptide myristoylated-EAVSLKPT (EAV; peptide sequence: H-Glu-Ala-Val-Ser-Leu-Lys-Pro-Thr-OH, 10 μM, Santa Cruz) were added in the culture medium at 10 min (30 min for EAV) before EtOH or vehicle exposure, and were then also included during EtOH exposure and withdrawal periods. All reagents were purchased from Sigma-Aldrich unless otherwise specified.

### Plasmids, siRNAs and Transfection

Rat GABA_A_R α1 and δ subunit cDNAs were obtained from M. Wallner (University of California, Los Angeles, CA, USA; Wallner et al., 2003). The ORFs and part of the 3′ UTRs of GABA_A_R α1 and δ subunits were amplified by PCR and cloned into a vector pcDNA3.1 with CMV promoter. The primers used for GABA_A_R-δ subunit were 5′-CCCAAGCTTATGGACGCGCCC-3′ (a HindIII site at the 5′ end) and 5′-CGGGATCCCATGGCGTATGCCG-3′ (a BamHI site at the 3′ end). The primers used for GABA_A_R α1 subunit were 5′-TAGCTAGCTATGAGGAAAAGTCCAGGTCTG-3′ (a Nhe1 site at the 5′ end) and 5′-GGAATTCTTGATGTGGTGT GGGGGCTTTTAG-3′ primers (an EcoRI site at the 3′ end). The PCR product was separated by electrophoresis and then purified by gel extraction kit (Simgen). pcDNA3.1-GABA_A_R-δ and pcDNA3.1-GABA_A_R-α1 were constructed by using T_4_ DNA ligase (BioLabs) to ligate the isolated PCR product with the vector. To obtain the recombinant plasmid pcDNA3.1-GABA_A_R-δ-mCherry, the fluorescent protein coding sequence was amplified using 5′-CCCAAGCTTATGGACGCGCCC-3′ and 5′-CGGGATCCCATGGCGTATGCCG-3′ primers by PCR, and then inserted into the BamHI site at the C-terminus of pcDNA3.1-GABA_A_R-δ. For construction of the recombinant pcDNA3.1-GABA_A_R-α1-EGFP, EGFP coding sequence was amplified using 5′-CGGG ATCCATGGTGAGCAAGGGCGAGG-3′ and 5′-CGGGAT CCTCACTTGTACAGCTCGTCC-3′ primers, and then inserted into the BamHI site at the C-terminal of pcDNA3.1-GABA_A_R-α1. pcDNA3.1-mCherry and pcDNA3.1-EGFP were used as the control plasmids. All primers were purchased from Thermo Fisher Scientific. All plasmids were sequenced to ensure correct reading frame, orientation and sequence.

Two different siRNA sequence pairs from Thermo Fisher Scientific were used simultaneously to selectively inhibit PKCγ (GenBank accession number: NM_012628.1). The sequences are as follows: pair 1, 5′-GGAGGAGGGCGAGUAUUACAAUGUA-3′ and 5′-UACAUUGUAAUACUCGCCCUCCUCC-3′; pair 2, 5′-UCGGCAUGUGUAAAGAGAAUGUCUU-3′ and 5′-AAGACAUUCUCUUUACACAUGCCGA-3′. Two different siRNA sequence pairs from GenePharma were used simultaneously to selectively inhibit PKCδ (GenBank accession number: NM_133307.1). The sequences are as follows: pair 1, 5′-GCAUCUCCUUCAAUUCCUATT-3′ and 5′-UAGGAAUUGAAGGAGAUGCTT-3′; pair 2, 5′-GCAAGAAGAACAACGGCAATT-3′ and 5′-UUGCCGUUGUUCUUCUUGCTT-3′. Scrambled siRNAs (5′-UUCUCCGAAGGUGUCACGUTT-3′ and 5′-ACGUGACACGUUCGGAGAATT-3′) were used as the negative controls.

Neurons at DIV5 were transfected with purified plasmids or siRNAs using lipofectamine 3000 in an Opti-MEM medium according to the vendor’s protocol. Briefly, neurons in 6-well plates were incubated with the plasmid DNA (2.5 μg/well) or siRNA (0.1 nM, 5 μl/well) combined with Lipofectamine 3000 (3.75 μl). Each transfection was done in triplicate. The media containing complexes were removed at 3.5–4 h after transfection, and the cells were rinsed twice with Opti-MEM medium and then refilled with half fresh Neurobasal culture medium plus half original medium. The siRNA transfected-neurons at DIV9–10 (for confocal microscopy) and the plasmid transfected-neurons at DIV13–14 (for biochemical analyses) were used. All reagents were purchased from Thermo Fisher Scientific unless otherwise specified.

### Whole-Cell Patch-Clamp Recording

Immediately before electrophysiological recording, neurons at DIV13–14 were transferred to a perfusion chamber and visualized with an inverted microscope (IX51; Olympus). Whole-cell patch-clamp recordings were performed in the voltage-clamp mode in putative pyramidal neurons (with an oval or pyramidal bright soma) at room temperature (22–23°C) at a holding potential of −70 mV. For GABA_A_R-mediated mIPSC recordings, neurons were perfused with a bath solution containing (in mM): 137 NaCl, 5 KCl, 2 CaCl_2_, 1 MgCl_2_, 20 Glucose and 10 HEPES (pH 7.40, 310–320 mOsm). Glass pipettes were filled with internal solution containing (in mM): 137 CsCl, 2 MgCl_2_, 1 CaCl_2_, 11 EGTA, 10 HEPES and 3 ATP (290–300 mOsm, pH adjusted to 7.30 with CsOH), with an input resistance of 4–7 MΩ. mIPSCs were pharmacologically isolated by adding tetrodotoxin (TTX, 0.5 μM, Ascent Scientific), APV (40 μM, Ascent Scientific), CNQX (10 μM, Ascent Scientific) and CGP54626 (1 μM, Tocris Bioscience) to the bath solution. Final concentrations of dimethyl sulfoxide (DMSO) did not exceed 0.01% in the recording chamber. All reagents were from Sigma-Aldrich unless specified otherwise. All functional recordings in drug-exposed neurons were carried out between 20 min and 40 min of drug incubation, and recordings for EtOH-withdrawn neurons were performed between 20 min and 40 min after EtOH withdrawal.

Recordings were amplified using an Axopatch 200B amplifier (Molecular Devices). Series resistance was normally less than 20 MΩ and recordings exceeding 20% change in series resistance were terminated and discarded. Electrophysiological recordings were filtered at 2.0 kHz and digitized at 50 kHz. Individual events were counted and analyzed with MiniAnalysis (Synaptosoft). For kinetic analysis, only single-event mIPSCs with a stable baseline, sharp rising phase (10%–90% rise time), and exponential decay were chosen during visual inspection of the recording traces. Double- and multiple-peak mIPSCs were excluded. The *I*_tonic_ magnitudes were obtained from the averaged baseline current of a given recording period. The amplitude of the *I*_tonic_ was calculated by the outward shift of the baseline holding currents after the application of bicuculline (10 μM), a competitive inhibitor of GABA_A_Rs, which can diminish both synaptic and *I*_tonic_ magnitude (Olsen and Sieghart, 2008; Shen et al., 2011). Only current recordings that exhibited a stable baseline were included in the analysis.

### Biotinylation Assay for Cell-Surface Protein of GABA_A_R-δ Subunits

Cultured neurons at DIV13–14 were placed on ice and rinsed twice with ice-cold phosphate-buffered saline (PBS). The neurons were then incubated for 20 min on ice with PBS containing 1 mg/ml sulfo-NHS-LC-biotin (ProteoChem). After rinsing with Tris-buffered saline (TBS) to quench the biotin reaction, neurons were lysed in 150 μl of RIPA buffer [1% Triton X-100, 0.1% sodium dodecyl sulfate (SDS), 150 mM NaCl, 2 mM EDTA, 50 mM NaF, 10 mM sodium pyrophosphate, 1.0 mM Na_3_VO_4_, 1.0 mM PMSF, and a complete protease inhibitor cocktail (Roche)]. The homogenates were centrifuged for 14,000 *g* × 15 min at 4°C. An aliquot (10%) of the supernatant was removed to measure β-actin. The remaining supernatant was incubated with 60 μl of 50% neutravidin agarose (Pierce) for 4 h at 4°C and washed four times with the lysis buffer. Agarose-bound proteins were taken up in 40 μl of SDS sample buffer and boiled (Shen et al., 2011).

### Western Blotting

Cell-surface protein levels of GABA_A_R-δ subunits and total protein levels of PKC isoforms were measured. Cultured neurons at DIV13–14 were washed twice with ice-cold PBS after the medium was carefully aspirated, and then were collected on ice with ice-cold RIPA buffer mentioned above in “Biotinylation Assay” section. The lysate was then centrifuged at 14,000 *g* × 15 min at 4°C and the supernatant collected for Western blot analysis. Protein concentrations were determined using a Pierce™ BCA Protein Assay Kit (Thermo Fisher Scientific) and the samples were stored at −20°C. Ten percentage SDS-PAGE was used to separate protein samples. After electrophoresis, the gels were transferred onto polyvinylidene fluoride (PVDF) membranes (Merck Millipore) using a constant voltage of 300 mA for 90 min. The membranes were then blocked in 5% milk in TBST (25 mM Tris-HCl, 150 mM NaCl and 0.1% Tween 20, pH 7.4) for 1.5 h at room temperature on a shaker, and incubated with the primary antibody overnight at 4°C. Primary antibodies were prepared with 5% milk in TBST [rabbit anti-GABA_A_R-δ (gift from Dr. W. Sieghart, Medical University Vienna, Austria), mouse anti-PKCδ (BD Biosciences), mouse anti-PKCε (BD Biosciences), rabbit anti-PKCγ (Abcam), and mouse anti-β-actin (Sigma-Aldrich)]. Next, the membranes were rinsed three times in TBST for 10 min each before incubation with an anti-mouse or anti-rabbit horseradish peroxidase (HRP)-conjugated secondary antibody (Thermo Fisher Scientific) for 1 h at room temperature. After being washed in TBST five times for 10 min each at room temperature, protein bands were visualized using ECL or ECL-Plus Western blotting detection reagents (Pierce). Quantification of the intensity of the protein bands was performed using the NIH ImageJ software. Surface protein amount was calculated by the optical density of each cell-surface subunit signal divided by optical density of the corresponding β-actin signal of the total cell lysate (% of β-actin) and compared to control that is set to 100% (Shen et al., 2016).

### Confocal Microscopy

Transfected-neurons at DIV9–10 were used for time-lapse live-cell imaging. The neurons in confocal dishes were perfused with bath solution containing (in mM): 145 NaCl, 5 KCl, 2 CaCl_2_, 2 MgCl_2_, 10 Glucose and 10 HEPES (pH 7.40, 290–310 mOsm) at 35°C. After baseline data (F_0_) were taken, the control bath solution was changed to bath solution containing 60 mM EtOH or drugs to record treated data (F). Then EtOH was washed out by normal culture medium, followed by another image taken at 30 min after withdrawal (F). The images were acquired by a Nikon A1 laser-scanning confocal microscope under a fixed set of settings. Images were viewed through a 60× oil-immersion objective (numerical aperture 1.4) and taken at 1024 × 1024 pixels resolution. The gain, threshold and offset levels were set equal during individual experiments (488 nm: gain, 7.00, HV, 50, offset, 0; 561 nm: gain, 1.70, HV 25, offset, 0). Pictures were analyzed by MetaMorph as previously described (Shen et al., 2016). Images were initially acquired as 12-bit grayscale and were prepared for presentation using Adobe Photoshop (Adobe Systems). The relative fluorescent signals expressed in arbitrary units (F/F_0_) were analyzed for individual cells using MetaMorph with a fixed set of parameters. All image analyses were done blind to the experimental condition. The numbers (n) in figure legends represent cell numbers from three independent cultures.

### Statistics

SigmaPlot, SigmaStat (Systat Software, Inc.) and Prism (GraphPad Software) were used for data display and statistical analysis. Significance is reported as *p* < 0.05, and data are expressed as mean ± SEM. Student *t* test, one-way analysis of variance (ANOVA) followed by a *post hoc* multiple comparison analysis based on the Dunnett’s method, or two-way ANOVA followed by a *post hoc* multiple comparison analysis based on the Holm-Sidak method were used to determine significant levels between treatments and controls.

## Results

### Protein Phosphorylation Contributed to the Down-Regulation of Extrasynaptic GABA_A_R-Mediated *I*_tonic_ by a Single EtOH Withdrawal

**Figure 1:**
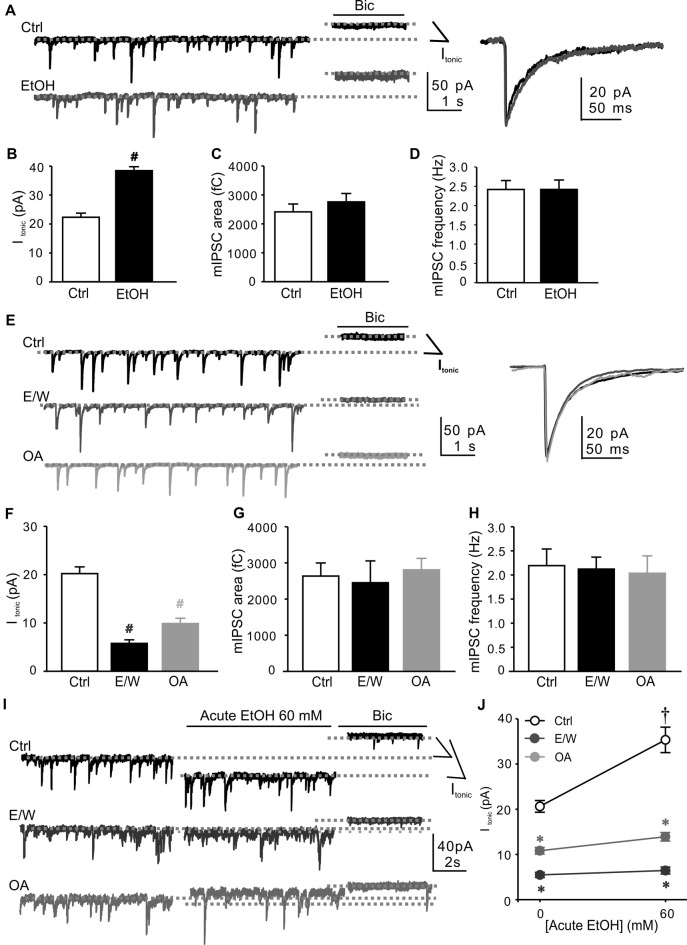
Okadaic acid (OA) mimics a single ethanol (EtOH) withdrawal-induced decreases in extrasynaptic GABA_A_R-mediated tonic current (*I*_tonic_) and *I*_tonic_ responsiveness to acute EtOH in cultured hippocampal neurons. Neurons were whole-cell voltage-clamped at −70 mV (holding potential, *I*_hold_). **(A)** Left: sample traces of individual recordings from neurons during 30-min vehicle (Ctrl) or 60 mM EtOH exposure (EtOH). Right: averaged miniature inhibitory postsynaptic currents (mIPSCs). The *I*_tonic_ amplitude is measured as currents at the experimental conditions vs. the mean current following bicuculline (Bic, 10 μM) application (dotted lines indicate *I*_hold_: basal, light-gray line commencing at left margin; Bic application, and light-gray line commencing near far right end). **(B–D)** Changes in *I*_tonic_ amplitude **(B)**, total chargetransfer of mIPSCs (mIPSC area, **C**) and mIPSC frequency **(D)** recorded in neurons from Ctrl and EtOH groups as shown in **(A)**. **(E)** Left: sample traces of individual recordings from neurons at 30 min after withdrawal from Ctrl and EtOH (60 mM for 30 min, E/W) as well as during 30-min OA (100 nM, OA) exposure. Right: averaged mIPSCs. **(F–H)** Changes in *I*_tonic_ amplitude **(F)**, mIPSC area **(G)** and frequency **(H)** recorded in neurons from Ctrl, E/W and OA groups as shown in **(E)**. **(I)** Sample traces of individual recordings from Ctrl-, E/W- and OA-treated neurons. The *I*_hold_ after acute EtOH application is indicated by light-gray dotted lines (middle line commencing near far right end). **(J)** The effects of EtOH on *I*_tonic_ amplitude from the same recordings as shown in **(I)**. *n* = 10–13 per group. ^#^*p* < 0.001 vs. Ctrl group (*t* test in **B** and one-way ANOVA with *post hoc* Dunnett’s test in **F**); ^†^*p* < 0.001 vs. the pre-EtOH baseline value, *p < 0.001 vs. Ctrl group (two-way ANOVA with *post hoc* Holm-Sidak test).

We first measured the miniature inhibitory postsynaptic currents (mIPSCs) and baseline tonic activity in cultured hippocampal neurons at DIV14 with 30-min vehicle (Ctrl) or 60 mM EtOH exposure. The concentration of 60 mM EtOH used to treat cultured neurons was chosen as previously reported (Shen et al., 2011) to match blood levels measured in adult rats after intoxication with gavage of 5 g/kg, which induced substantial plasticity in GABA_A_Rs and drug tolerance (Liang et al., 2007). EtOH exposure for 30 min greatly potentiated *I*_tonic_ amplitude (*t* test; *t* = −8.662, *p* < 0.001; Figures 1A,B), with no effect on either mIPSC area (Figures 1A,C) or frequency (Figures 1A,D). However, 30-min withdrawal from a single EtOH exposure (E/W) induced a significant reduction in *I*_tonic_ amplitude (one-way ANOVA; *F*_(2,33)_ = 84.586, *p* < 0.001 vs. Ctrl group; Figures 1E,F). Virtually no change in either mIPSC area (Figures 1E,G) or frequency (Figures 1E,H) was observed in E/W-neurons compared with Ctrl group. The finding indicates that a single EtOH withdrawal, but not EtOH exposure, reduces extrasynaptic GABA_A_R function in neurons. To determine whether protein phosphorylation plays a role in a single EtOH withdrawal regulation of GABA_A_Rs, we also examined *I*_tonic_ amplitude and mIPSCs in neurons treated with a phosphatase inhibitor OA. Similar results were observed in OA-treated (100 nM, 30 min) neurons in the absence of EtOH (one-way ANOVA; *F*_(2,33)_ = 84.586, *p* < 0.001 vs. Ctrl group; Figures 1E–H) compared with the neurons in E/W group.

Since extrasynaptic δ-GABA_A_Rs are thought to be particularly and highly sensitive for EtOH (Olsen et al., 2007; Shen et al., 2011), next we tested the *I*_tonic_ responsiveness to acute EtOH in Ctrl-, E/W- and OA-neurons. Approximately 80% of the control neurons recorded (*n* = 13) showed *I*_tonic_ potentiation by acute EtOH (60 mM; two-way ANOVA; *F*_(1,66)_ = 32.589, *p* < 0.001 vs. pre-EtOH baseline value; Figures 1I,J), consistent with our previous study (Shen et al., 2011). However, 30-min withdrawal from both a single EtOH and OA exposure resulted in *I*_tonic_ tolerance to acute EtOH (two-way ANOVA; *F*_(2,66)_ = 150.933, *p* < 0.001 vs. Ctrl group; *F*_(2,66)_ = 15.826, *p* < 0.001, significant interaction effect between groups and E/W treatments; Figures 1I,J), suggesting that protein phosphorylation contributes to the rapid down-regulation of extrasynaptic δ-GABA_A_R sensitivity to acute EtOH by a single EtOH withdrawal.

### PKC Inhibition Prevented the Decrease in *I*_tonic_ Amplitude and Acute EtOH Sensitivity Induced by a Single EtOH Withdrawal

**Figure 2:**
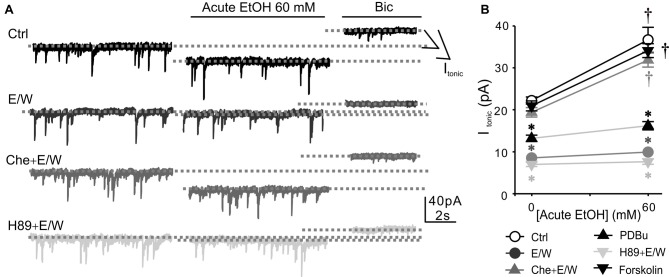
Protein kinase C (PKC) inhibition prevents a single EtOH withdrawal-induced decrease in *I*_tonic_ amplitude and acute EtOH responsiveness. **(A)** Sample traces of individual recordings from neurons obtained at 30 min after withdrawal from Ctrl, EtOH (E/W), chelerythrine chloride (Che, 10 μM) plus EtOH (Che+E/W), and H89 (20 μM) plus EtOH (H89+E/W) exposure. **(B)** Changes in *I*_tonic_ amplitude and acute EtOH responsiveness in neurons from Ctrl, E/W, Che+E/W, phorbol 12, 13-dibutyrate (PDBu; 100 nM, 30 min), H89+E/W, and forskolin (10 μM, 30 min) groups. *n* = 10–14 per group. ^†^*p* < 0.001 vs. the pre-EtOH baseline value, *p < 0.001 vs. Ctrl group (two-way analysis of variance (ANOVA) with *post hoc* Holm-Sidak test).

It has been reported that PKC and protein kinase A (PKA) play important but differential roles in facilitating GABA_A_Rs after EtOH exposure (Kumar et al., 2010, 2012; Werner et al., 2011; Bohnsack et al., 2016; Carlson et al., 2016a,b). Therefore, we next examined the impact of inhibiting PKC or PKA in the presence of EtOH to determine whether PKC or PKA might be involved in the effects of a single EtOH withdrawal on extrasynaptic δ-GABA_A_Rs. Inhibiting PKC by chelerythrine chloride (Che, 10 μM) in the presence of EtOH prevented a single EtOH withdrawal-induced decreases in *I*_tonic_ amplitude and acute EtOH sensitivity (two-way ANOVA; *F*_(5,132)_ = 104.6, *p* < 0.001 vs. Ctrl group; *F*_(1,132)_ = 95.471, *p* < 0.001 vs. pre-EtOH baseline value; *F*_(5,132)_ = 11.608, *p* < 0.001, significant interaction effect between groups and E/W treatments; Figure 2). However, PKA inhibition by H89 (20 μM) had no effect on EtOH withdrawal-regulation of *I*_tonic_ amplitude and acute EtOH tolerance. Che or H89 alone had no effect on either *I*_tonic_ amplitude or acute EtOH responsiveness (Table 1). The results indicated that a single EtOH withdrawal-induced *I*_tonic_ reduction and acute EtOH tolerance were prevented by PKC but not PKA inhibition. We then used the PKC activator PDBu (100 nM) to determine whether PKC activation has similar activity in our system. PDBu exposure in the absence of EtOH resulted in smaller but significant *I*_tonic_ reduction and acute EtOH tolerance, consistent with the results observed in the OA group. However, PKA activation by forskolin (10 μM) in the absence of EtOH had no effect on either *I*_tonic_ amplitude or acute EtOH responsiveness. No alteration in mIPSC area or frequency was observed in the experiments (Table 1). Taken together, these results suggest that PKC but not PKA activation contributes to the rapid down-regulation of extrasynaptic δ-GABA_A_Rs by a single EtOH withdrawal.

**Table 1:** Effects of ethanol (EtOH) and drug treatments on *I*_tonic_ amplitude, mIPSC area and mIPSC frequency.

	*I*_tonic_ amplitude (pA)		mIPSC area (fC)		mIPSC Frequency (Hz)	
	Baseline	Acute EtOH	Baseline	Acute EtOH	Baseline	Acute EtOH
Ctrl	22.2 ± 0.9	36.6 ± 3.0^†^	2995.3 ± 232.5	2705.1 ± 193.7	2.47 ± 0.18	2.54 ± 0.21
E/W	8.5 ± 0.5	9.9 ± 0.7*	2615.5 ± 276.5	2648.3 ± 211.7	2.56 ± 0.23	2.65 ± 0.21
Che + E/W	18.9 ± 1.1	31.8 ± 1.7^†^	2895.7 ± 254.6	2791.6 ± 305.9	2.36 ± 0.16	2.45 ± 0.15
Che	20.8 ± 1.5	33.3 ± 2.2^†^	2811.4 ± 297.9	2855.4 ± 224.1	2.49 ± 0.26	2.62 ± 0.24
PDBu + E/W	6.1 ± 0.6	6.9 ± 0.6*	2508.3 ± 226.2	2811.1 ± 297.9	2.28 ± 0.23	2.32 ± 0.23
PDBu	13.1 ± 0.7	16.1 ± 0.8*	2805.8 ± 258.2	2592.9 ± 232.5	2.45 ± 0.20	2.57 ± 0.18
H89 + E/W	6.9 ± 0.5	7.6 ± 0.6*	2682.0 ± 312.7	2772.3 ± 148.4	2.32 ± 0.16	2.43 ± 0.20
H89	20.0 ± 0.7	31.3 ± 1.8^†^	2888.2 ± 208.5	2801.8 ± 225.0	2.60 ± 0.12	2.67 ± 0.17
Forskolin + E/W	7.0 ± 0.8	8.2 ± 0.9*	2662.5 ± 200.7	2504.7 ± 185.3	2.23 ± 0.18	2.35 ± 0.17
Forskolin	20.9 ± 1.2	34.1 ± 1.7^†^	2516.8 ± 150.2	2690.0 ± 219.5	2.16 ± 0.24	2.15 ± 0.17

n = 11–14 per group. ^†^p < 0.001 vs. the pre-EtOH baseline value, *p < 0.001 vs. Ctrl group (two-way ANOVA with post hoc Holm-Sidak test).

### PKC Inhibition Prevented the Reduction in Cell-Surface Level of Extrasynaptic δ Subunits by a Single EtOH Withdrawal

**Figure 3:**
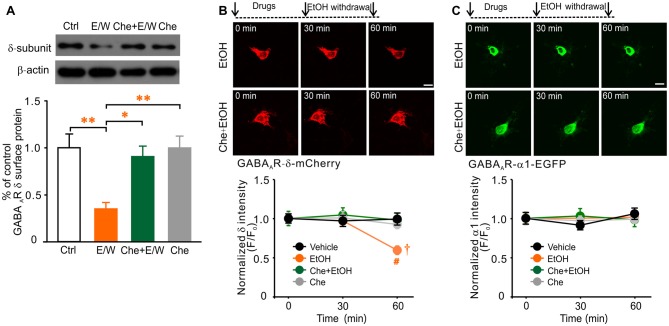
PKC inhibition prevents a single EtOH withdrawal-induced reduction in cell-surface level of native GABA_A_R-δ subunits and total protein level of recombinant δ subunits. **(A)** Representative western blots (top panels) and corresponding quantification (lower panel) of the biotinylation assay for cell-surface GABA_A_R-δ level in neurons from Ctrl, E/W, Che+E/W, and Che groups. β-actin of total cell lysate was used as the loading control. *n* = 4–5 per group. **(B)** Representative time-lapse live-cell images of pcDNA3.1-GABA_A_R-δ-mCherry transfected-neurons from EtOH and Che+EtOH treatments (top panels) and corresponding quantification of changes in recombinant δ subunit intensity in neurons from vehicle, EtOH, Che+EtOH, and Che treatments, respectively (lower panel). **(C)** Representative time-lapse live-cell images of pcDNA3.1-GABA_A_R-α1-EGFP transfected-neurons from EtOH and Che+EtOH treatments (top panels) and corresponding quantification of recombinant α1 subunit intensity in neurons from vehicle, EtOH, Che+EtOH, and Che treatments, respectively (lower panel). Scale bars = 10 μm. *n* = 19–21 per group. *p < 0.05, ^**^p < 0.01 (one-way ANOVA with *post hoc* Dunnett’s test); ^†^*p* < 0.05 vs. the pre-drug baseline value, ^#^*p* < 0.05 vs. vehicle group (two-way ANOVA with *post hoc* Holm-Sidak test).

Because extrasynaptic sensitivity to EtOH is correlated to δ subunit expression in cultured neurons (Shen et al., 2011), it is possible that the cell-surface level of δ subunits may be also altered in our cultures. Biotinylation assay and western blot analysis revealed that a single EtOH withdrawal notably reduced the cell-surface level of δ content (one-way ANOVA; *F*_(3,18)_ = 7.428, *p* = 0.003; Figure 3A), whereas 30-min EtOH exposure had no effect (data not shown). These results illustrated that an EtOH withdrawal reduced both δ-GABA_A_R function and δ subunit level in hippocampal neurons as previously reported (Shen et al., 2011). Withdrawal from EtOH exposure in the presence of Che prevented the reduction in cell-surface δ abundance (*p* = 0.932, Che+E/W vs. Ctrl group; *p* = 0.016, Che+E/W vs. E/W group), while Che alone had no effect (*p* = 0.999, Che vs. Ctrl group; *p* = 0.009, Che vs. E/W group; Figure 3A). We then measured the fluorescence intensity of recombinant δ subunit in neurons transfected with the pcDNA3.1-GABA_A_R-δ-mCherry plasmid or control plasmid by time-lapse live-cell imaging. A single EtOH withdrawal, but not exposure, reduced the fluorescent intensity of recombinant δ subunits (two-way ANOVA; *F*_(2,231)_ = 3.888, *p* = 0.022 vs. pre-drug baseline value; Figure 3B). No alteration of recombinant δ intensity in vehicle group was observed during the whole period. PKC inhibition by Che blocked EtOH withdrawal-induced reduction in recombinant δ intensity (two-way ANOVA; *F*_(3,231)_ = 2.675, *p* = 0.048 vs. vehicle group; *F*_(6,231)_ = 2.229, *p* = 0.041, significant interaction effect between groups and drug treatments; Figure 3B). Together, these data indicate that the down-regulation of extrasynaptic δ-GABA_A_Rs by a single EtOH withdrawal is most likely mediated by PKC activation.

Since typical synaptic GABA_A_R-α1 subunits (Rudolph et al., 2001) have reported to be down-regulated by EtOH (Liang et al., 2007; Kumar et al., 2009), we then determined the fluorescence intensity of recombinant α1 subunits in neurons transfected with the pcDNA3.1-GABA_A_R-α1-EGFP plasmid. No alteration in recombinant α1 intensity was found in neurons treated with EtOH exposure and withdrawal, Che co-exposure with EtOH and withdrawal or Che alone (Figure 3C), consistent with previously reported that the regulation of synaptic GABA_A_R-α1 subunits by a single EtOH withdrawal does not occur at early time point (within 1 h) after withdrawal (Shen et al., 2011). Meanwhile, PKC activation might not be involved in the regulation of α1 subunits by short-term (30 min) EtOH exposure or withdrawal.

### PKCδ Inhibition Prevented the Reductions in Cell-Surface Protein Level of δ Subunits and δ-GABA_A_R Function Induced by a Single EtOH Withdrawal

**Figure 4:**
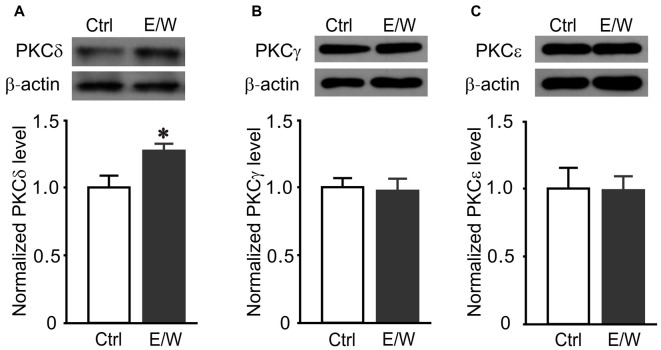
A single EtOH withdrawal selectively increases total protein levels of PKCδ isoform in cultured neurons. **(A–C)** Representative western blots (top panels) and corresponding quantification (lower panels) of total protein levels of PKCδ **(A)**, PKCγ **(B)** and PKCε **(C)** isoforms in neurons from Ctrl and E/W groups, respectively. *n* = 4–5 per group. *p < 0.05 (*t* test).

Since the effects of a single EtOH withdrawal on δ-GABA_A_Rs were partially mimicked by PKC activation and blocked by PKC inhibition, we further investigated whether specific PKC isoforms were involved in these effects. Thirty-minute withdrawal from a single EtOH exposure increased PKCδ (*t* test; *t* = −2.678, *p* = 0.028; Figure 4A) but not PKCγ (Figure 4B) or PKCε level (Figure 4C) in hippocampal neurons, indicating that PKCδ may contribute to a single EtOH-withdrawal regulation of δ-GABA_A_Rs.

**Figure 5:**
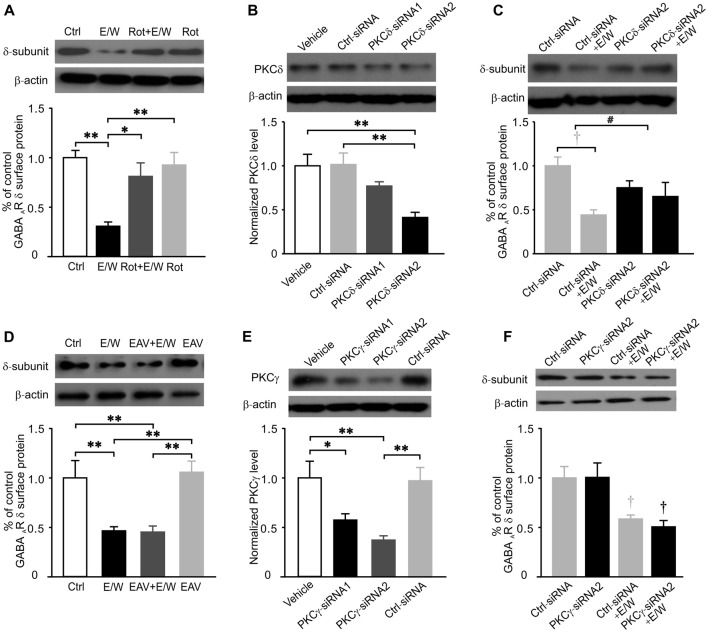
PKCδ inhibition prevents the reduction in cell-surface GABA_A_R-δ level by a single EtOH withdrawal. **(A)** Representative western blots (top panels) and corresponding quantification (lower panel) of the biotinylation assay for cell-surface GABA_A_R-δ levels in neurons from Ctrl, E/W, PKCδ inhibitor rottlerin plus EtOH (Rot+E/W), and Rot alone treatments. **(B)** Representative western blots (top panels) and corresponding quantification (lower panel) of total protein levels of PKCδ isoform in neurons from vehicle, negative control siRNA (Ctrl-siRNA), PKCδ-siRNA1 and PKCδ-siRNA2 groups, respectively. **(C)** Representative western blots (top panels) and corresponding quantification (lower panel) of cell-surface GABA_A_R-δ levels in neurons from Ctrl-siRNA, PKCδ-siRNA2, Ctrl-siRNA+E/W, and PKCδ-siRNA2+E/W treatments, respectively.** (D)** Representative western blots (top panels) and corresponding quantification (lower panel) of cell-surface GABA_A_R-δ levels in neurons from Ctrl, E/W, PKCε inhibitor peptide EAVSLKPT plus EtOH (EAV +E/W) and EAV alone treatments, respectively. **(E)** Representative western blots (top panels) and corresponding quantification (lower panel) of total protein levels of PKCγ isoform in neurons from vehicle, Ctrl-siRNA, PKCγ-siRNA1 and PKCγ-siRNA2 groups, respectively. **(F)** Representative western blots (top panels) and corresponding quantification (lower panel) of cell-surface GABA_A_R-δ levels in neurons from Ctrl-siRNA, PKCγ-siRNA2, Ctrl-siRNA+E/W, and PKCγ-siRNA2+E/W treatments, respectively. *n* = 4–6 per group. *p < 0.05, ^**^p < 0.01 (one-way ANOVA with *post hoc* Dunnett’s test); ^#^*p* < 0.05, PKCδ knockdown vs. Ctrl-siRNA groups, ^†^*p* < 0.01, E/W vs. non-E/W group (two-way ANOVA with *post hoc* Holm-Sidak test).

**Figure 6:**
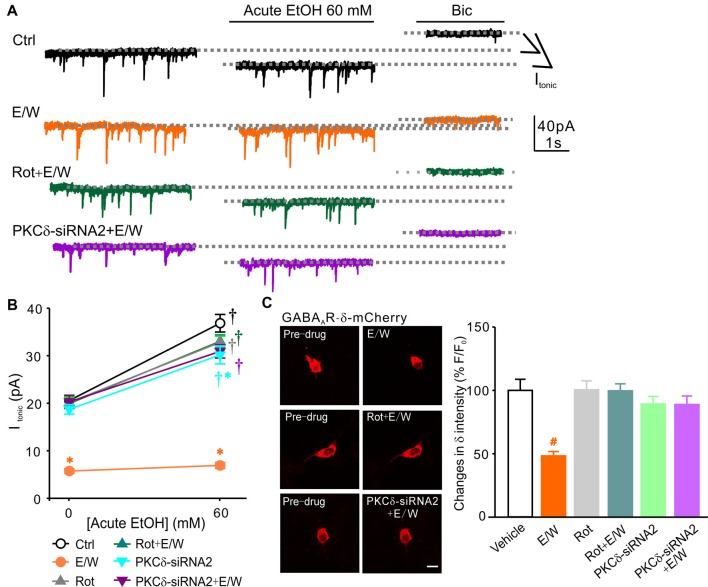
PKCδ inhibition prevents the reductions in extrasynaptic δ-GABA_A_R function and δ subunit level by a single EtOH withdrawal. **(A)** Sample traces of individual recordings in neurons from Ctrl, E/W, Rot+E/W and PKCδ-siRNA2+E/W treatments. **(B)** Changes in acute EtOH responsiveness in neurons from Ctrl, E/W, Rot, Rot+E/W, PKCδ-siRNA2, and PKCδ-siRNA2+E/W treatments, respectively. *n* = 10–14 per group. **(C)** Representative time-lapse live-cell images of pcDNA3.1-GABA_A_R-δ-mCherry transfected-neurons from E/W, Rot+E/W and PKCδ-siRNA2+E/W treatments (left panels) and changes in recombinant δ subunit intensity in transfected-neurons from vehicle, E/W, Rot, Rot+E/W, PKCδ-siRNA2, and PKCδ-siRNA2+E/W treatments, respectively (right panel). Scale bar = 10 μm. *n* = 20 per group. ^†^*p* < 0.001 vs. the pre-EtOH baseline value (black ^†^, Ctrl; gray ^†^, Rot; green ^†^, Rot+E/W; cyan ^†^, PKCδ-siRNA2; purple ^†^, PKCδ-siRNA2+E/W), *p < 0.001 vs. Ctrl group (cyan *, PKCδ-siRNA2; orange *, E/W; two-way ANOVA with *post hoc* Holm-Sidak test); ^#^*p* < 0.001, E/W vs. vehicle group, Rot+E/W vs. E/W group, or PKCδ-siRNA2+E/W vs. E/W group (one-way ANOVA with *post hoc* Dunnett’s test).

To determine whether a single EtOH withdrawal-induced reduction in cell-surface protein level of δ subunits is specifically dependent on PKCδ activation, selective PKCδ inhibitor Rottlerin (Rot, 10 μM) and specific PKCδ siRNAs were both used. EtOH withdrawal in the presence of Rot (Rot+E/W) failed to induce any reduction in cell-surface level of δ subunits (one-way ANOVA; *F*_(3,18)_ = 8.173, *p* = 0.002; *p* = 0.003, E/W vs. Ctrl group; *p* = 0.608, Rot+E/W vs. Ctrl group; *p* = 0.015, Rot+E/W vs. E/W group), while Rot alone had no effect (*p* = 0.966, Rot vs. Ctrl group; *p* = 0.007, Rot vs. E/W group; Figure 5A). The PKCδ siRNAs reduced PKCδ levels by approximately 25% (PKCδ-siRNA1) and 60% (PKCδ-siRNA2), respectively, compared to vehicle-neurons (one-way ANOVA; *F*_(3,12)_ = 7.917, *p* = 0.004; *p* = 0.006, PKCδ-siRNA2 vs. vehicle group; *p* = 0.005; PKCδ-siRNA2 vs. Ctrl-siRNA group; Figure 5B). No significance was found between negative control siRNA (Crtl-siRNA) and vehicle groups. A single EtOH withdrawal induced a significant decrease in GABA_A_R-δ surface protein; in the condition of PKCδ-siRNA2, EtOH withdrawal did not induced significant effects (two-way ANOVA; *F*_(1,20)_ = 5.087, *p* = 0.035, PKCδ knockdown vs. Ctrl-siRNA groups; *F*_(1,20)_ = 10.246; *p* = 0.004, E/W vs. non-E/W group; *F*_(1,20)_ = 2.437; *p* = 0.048, significant interaction effect between groups and E/W treatments; Figure 5C), suggesting that EtOH withdrawal-induced reduction in cell-surface protein level of δ subunits depends on PKCδ activation. Besides, we also tested the roles of PKCε and PKCγ inhibition on EtOH withdrawal-induced reduction in GABA_A_R-δ abundance. PKCε inhibition by inhibitory peptide EAVSLKPT (EAV) in the presence of EtOH had no effect on EtOH withdrawal-induced reduction in cell-surface level of δ subunits (one-way ANOVA; *F*_(3,17)_ = 12.382, *p* = 0.001; *p* = 0.009, E/W vs. Ctrl group; *p* = 0.008, EAV+E/W vs. Ctrl group; *p* = 0.004, EAV vs. E/W group; *p* = 0.003, EAV vs. EAV+E/W group; Figure 5D). PKCγ inhibition by specific PKCγ siRNAs, which significantly reduced PKCγ levels (one-way ANOVA; *F*_(3,16)_ = 7.319, *p* = 0.003; *p* = 0.046, PKCγ-siRNA1 vs. vehicle group; *p* = 0.004, PKCγ-siRNA2 vs. vehicle group; *p* = 0.009, PKCγ-siRNA2 vs. Ctrl-siRNA group; Figure 5E), did not block the reduced cell-surface δ content (two-way ANOVA; *F*_(1,19)_ = 0.156, *p* = 0.698, PKCγ knockdown vs. Ctrl-siRNA groups; *F*_(1,19)_ = 23.422, *p* < 0.001; Figure 5F). PKCδ inhibition by both Rot and PKCδ-siRNA2 prevented EtOH withdrawal-induced reduction in *I*_tonic_ amplitude and acute EtOH tolerance (two-way ANOVA; *F*_(5,140)_ = 80.767, *p* < 0.001 vs. Ctrl group; *F*_(1,140)_ = 218.169, *p* < 0.001 vs. the pre-EtOH baseline value; *F*_(5,140)_ = 7.620, *p* < 0.001, significant interaction effect between groups and E/W treatments; Figures 6A,B), although a slight reduction in *I*_tonic_ potentiation by acute EtOH in PKCδ-siRNA2 group was observed (*p* = 0.013, PKCδ-siRNA2 vs. Ctrl group; Figures 6A,B). In addition, both Rot and PKCδ-siRNA2 blocked EtOH withdrawal-induced reduction in recombinant δ intensity (one-way ANOVA; *F*_(5,114)_ = 9.650, *p* < 0.001; *p* < 0.001, E/W vs. vehicle group; *p* < 0.001, Rot+E/W vs. E/W group; *p* < 0.001, PKCδ-siRNA2+E/W vs. E/W group; Figure 6C). Taken together, these data suggest that a single EtOH withdrawal modulates extrasynaptic δ-GABA_A_R function and protein level of δ subunits via PKCδ activation.

## Discussion

The profound rapid reductions in extrasynaptic δ-GABA_A_R-mediated function and protein level of δ subunits in hippocampal neurons induced by a single EtOH withdrawal suggest that PKCδ plays an important role in the alteration of sensitivity to EtOH. Such alterations can be partially mimicked by a phosphotase inhibitor (OA) and a PKC activator (PDBu) exposure but was successfully blocked by the general PKC inhibitor (Che), selective PKCδ inhibitor (Rot) and specific PKCδ siRNAs. The findings provide direct evidence that a single EtOH withdrawal rapidly regulates extrasynaptic δ-GABA_A_Rs via PKCδ activation.

Previous studies have reported that the rat model of CIE and withdrawal simulates the symptoms of EtOH dependence and EtOH withdrawal in humans, including reduced seizure threshold and enhanced seizure susceptibility that related to GABA_A_R alterations (Olsen and Spigelman, 2012). GABA_A_R subunit composition, function, and sensitivity to EtOH and benzodiazepines are altered in CIE-rats, accompanied by behavior abnormalities (Liang et al., 2006). A single intoxicating EtOH exposure could induce similar but temporary GABA_A_R changes (known as GABA_A_R plasticity) *in vivo* (Liang et al., 2007), while a single EtOH withdrawal leads to similar alterations *in vitro* (Shen et al., 2011). A single EtOH exposure triggers a short and small withdrawal response (mini-withdrawal) at every EtOH intake, and the single “mini-withdrawal” results in GABA_A_R plastic changes. Chronic EtOH exposure may further induce repeated “mini-withdrawals” to keep the brain in a continuous state of withdrawal, resulting in irreversible and permanent pathological changes from temporary GABA_A_R plasticity (Liang et al., 2007; Olsen and Spigelman, 2012; Olsen and Liang, 2017). Importantly, in addition to the remarkable GABA_A_R plasticity, the animals with a single intoxicating dose of EtOH exhibit tolerance to benzodiazepine- and high dose EtOH-induced loss of righting reflex (LORR; Liang et al., 2007). Therefore, it suggests that a single EtOH withdrawal-regulation of GABA_A_Rs may be the basis for chronic EtOH tolerance and alcohol withdrawal syndromes (AWS; Olsen and Liang, 2017).

Tonic inhibitory currents are mediated by extrasynaptic GABA_A_Rs, activated by ~0.2–2.0 μM ambient or “spillover” extracellular GABA (Mody and Pearce, 2004; Semyanov et al., 2004; Farrant and Nusser, 2005). Tonic inhibition is mediated primarily by α5βγ2-GABA_A_Rs in the hippocampal CA1 region (Caraiscos et al., 2004; Mangan et al., 2005) and by α4βδ-GABA_A_Rs in the dentate gyrus (Laurie et al., 1992; Sperk et al., 1997; Peng et al., 2002; Liang et al., 2006). Tonic inhibition is thought to play a more important role in controlling neuronal excitability rather than phasic inhibition (Mody and Pearce, 2004). The rapid (within 1-h withdrawal) internalization of extrasynaptic δ-GABA_A_Rs by a single EtOH exposure and withdrawal occurs in both the rat model and cultured neurons (Liang et al., 2007; Shen et al., 2011), leading to a rapid drop of *I*_tonic_ and acute EtOH tolerance. In comparison, synaptic α1-GABA_A_R internalization occurs at 4-h withdrawal, resulting in delayed decreases in synaptic inhibition and cross-tolerance to benzodiazepines (Shen et al., 2011). This demonstrates that extrasynaptic δ-GABA_A_Rs could be initial responders to a single EtOH withdrawal (Gonzalez et al., 2012). In our study, we observed a rapid reduction of *I*_tonic_ amplitude and acute EtOH tolerance by 30-min EtOH withdrawal, which were paralleled with the decreased protein level of δ subunits. Neither area nor frequency of mIPSCs was affected by EtOH withdrawal, consistent with unaltered protein level of α1 subunits. The results are similar to the findings reported previously (Shen et al., 2011). By contrast, 30-min EtOH exposure increased the baseline tonic activity with no effect on the protein level of δ subunits, similar as the effects found in acute EtOH (60 mM) application (Liang et al., 2007; Shen et al., 2011; Carlson et al., 2016b), indicating that 60 mM EtOH exposure for 30 min acts as acute EtOH challenge in our system. Although the exactly mechanism has yet to be clarified, it is possible that withdrawal from an intoxicating, acute EtOH exposure may accelerate EtOH regulation of extrasynaptic GABA_A_Rs in cultures *in vitro*. Additionally, a decrease in protein level of recombinant δ but not α1 subunits was also observed, supporting that 30-min EtOH withdrawal specifically down-regulated the protein level of GABA_A_R-δ subunits. The differences in timing of changes in extrasynaptic and synaptic GABA_A_Rs suggest the distinct mechanisms of responsiveness to EtOH, possibly due to the highly preferential sensitivity of extrasynaptic δ-GABA_A_Rs to EtOH (Sundstrom-Poromaa et al., 2002; Wallner et al., 2003). Besides, our prior work (Shen et al., 2011) shows that α5βγ2-GABA_A_R-mediated tonic currents are also affected by EtOH, with slightly delayed α5 internalization (does not occur at 20-min withdrawal, whereas α4 and δ do). These data suggest that α5βγ2-GABA_A_Rs are downstream, but not the initial responders from EtOH activation. Although we cannot rule out the contribution of α5βγ2-GABA_A_Rs to tonic inhibition in the current study, it is likely that α5βγ2-GABA_A_R alterations by EtOH may have a different mechanism of regulation.

In our cultures, 30-min OA exposure partially mimicked the effects of a single EtOH withdrawal on *I*_tonic_ amplitude and acute EtOH sensitivity, suggesting the important roles of protein phosphorylation in a single EtOH withdrawal-regulation of extrasynaptic δ-GABA_A_Rs. This is consistent with previous evidence that protein phosphorylation is crucial for EtOH regulation of both synaptic and extrasynaptic receptors (Qi et al., 2007; Choi et al., 2008; Jacob et al., 2008; Nakamura et al., 2015). PKC has been shown to play important roles in EtOH regulation of GABA_A_Rs. Our results showed that PKC inhibition prevented EtOH withdrawal-induced *I*_tonic_ reduction and acute EtOH tolerance as well as the reduced protein level of GABA_A_R-δ subunits, indicating that PKC activation contributes to a single EtOH withdrawal-regulation of extrasynaptic δ-GABA_A_Rs. These results are supported by the previous study showing that PKC activation causes down-regulation of tonic GABA_A_R-mediated inhibition in dentate gyrus granule cells in the hippocampus and dorsal lateral geniculate relay neurons in the thalamus (Bright and Smart, 2013). Furthermore, 30-min EtOH withdrawal selectively increased total protein level of PKCδ in cultures, which is not surprising since previous studies report that EtOH exposure alters the subcellular localization of PKCδ in neuronal cells (Messing et al., 1991; Gordon et al., 1997). Meanwhile, PKCδ inhibition specifically blocked the reductions in protein levels of both native and recombinant δ subunits, as well as acute EtOH tolerance. The data indicate that the rapid regulation of extrasynaptic δ-GABA_A_Rs by a single EtOH withdrawal is most likely mediated via PKCδ activation. However, the present results are not consistent with previous studies showing that EtOH enhancement of α4β3δ-GABA_A_R function is PKCδ-dependent (Choi et al., 2008) and PKC activity regulates α4-GABA_A_R membrane insertion by phosphorylation of the α4 subunits (Abramian et al., 2010). Although we do not know whether the differences in expression systems (neurons vs. non-neuron cell lines) or EtOH treatments (a single intoxicating EtOH withdrawal vs. acute EtOH response or non-EtOH application), both may contribute to the discrepant results. Besides, PKCβ, PKCγ and PKCε levels in cultured cortical neurons are also altered by EtOH exposure in a previous study (Kumar et al., 2010), however, no alteration in either PKCγ or PKCε level was observed in hippocampal neurons with a single EtOH withdrawal in our experiment. We postulate that it may be due to the different neuron types and EtOH treatment procedures.

PKA inhibition did not prevent either EtOH withdrawal-induced *I*_tonic_ reduction or acute EtOH tolerance, while PKA activation also had no effect on extrasynaptic δ-GABA_A_R function in our study. The data are not consistent with previous studies showing the major roles of PKA in EtOH regulation of extrasynaptic α4-GABA_A_R function and subunit expression both *in vivo* (Carlson et al., 2014) and *in vitro* (Carlson et al., 2016a,b), since there are paralleled changes in protein levels of α4 and δ subunits as well as α4δ-GABA_A_R function induced by EtOH withdrawal in neurons (Shen et al., 2011). Although we cannot rule out the possibility that short application (30 min) of forskolin alone may not have been long enough to detect changes in responding or may require EtOH activation synchronously, our present data suggest that PKA activation may not play a role in the condition of a single intoxicating EtOH withdrawal but not prolonged EtOH exposure (1–4 h) in previous studies (Carlson et al., 2016a,b).

Clathrin-dependent endocytosis is one of the major internalization mechanisms for neuronal GABA_A_Rs (Kittler et al., 2000). The clathrin adaptor protein 2 (AP2) complex plays a critical role in recruiting membrane associated proteins into clathrin-coated pits. Phosphorylation of GABA_A_R subunits at distinct AP2 binding sites can regulate the cell surface stability of GABA_A_Rs, the strength of synaptic inhibition (Jacob et al., 2008) and tonic inhibition (Kumar et al., 2009). Differential regulation of GABA_A_R subunits by phosphorylation may be one of the key factors contribute to the discrepant EtOH regulation of GABA_A_R function and expression. One previous study demonstrates that EtOH promotes AP2-mediated endocytosis via the intracellular domain of δ-GABA_A_Rs (δ-ICD; Gonzalez et al., 2012). However, neither the changes in β2/3 association with AP2 machinery caused by phosphorylation nor α4 phosphorylation is responsible for the endocytosis, since the dephosphorylation of β3 and γ2 subunits promotes internalization of synaptic GABA_A_Rs (Kittler et al., 2005) whereas α4 phosphorylation dictates insertion into the membrane (Abramian et al., 2010). The final possibility for the rapid endocytosis of δ-GABA_A_Rs is the primary interactions involving the δ subunits, as EtOH increases the affinity between δ-ICD and AP2-μ2 (Gonzalez et al., 2012). The rapid down-regulation of extrasynaptic δ-GABA_A_Rs by a single EtOH withdrawal in present study is likely triggered by the increased interaction between δ-ICD and AP2-μ2 via PKCδ-mediated phosphorylation of μ2 regions in δ subunits. One previous study suggests a phosphorylation regulation of GABA_A_R-α4 subunits in the thalamus by PKCγ and PKCδ isoforms (Werner et al., 2016), however, it is unclear whether δ-ICD can be directly phosphorylated by PKCδ or other PKC isoforms. It would be important to test the functional significance of these μ2 binding sites within the ICD, as they potentially regulate the cell surface number of δ-GABA_A_Rs (Gonzalez et al., 2012). Future studies are required to clarify the mechanisms of a single EtOH withdrawal-regulation of δ-GABA_A_Rs via phosphorylation by PKCδ or other PKC isoforms.

In conclusion, the current work demonstrates a crucial role for PKCδ in the rapid regulation of extrasynaptic δ-GABA_A_R function and protein level by a single EtOH withdrawal. In view of the hypothesis that extrasynaptic δ-GABA_A_R endocytosis is the possible trigger for EtOH-induced GABA_A_R plasticity, PKCδ-mediated protein phosphorylation is potentially an important mechanism underlying EtOH withdrawal-regulation of GABA_A_Rs. Therefore, selective targeting of PKC isoforms may serve as potentially therapeutic approaches that aim to restore normal GABA_A_R function and subunit abundance altered by EtOH exposure and withdrawal.

## Author Contributions

YS conceived the project with contributions by RWO and JL. YS and JC designed the experiments. JC, YH, YW, HZ, L-DS and W-NL conducted the experiments. JC, YH and HZ analyzed the data. YS and Y-DZ wrote the manuscript. All authors contributed to the critical revision of the data and read and approved the final manuscript.

## Conflict of Interest Statement

The authors declare that the research was conducted in the absence of any commercial or financial relationships that could be construed as a potential conflict of interest.

## Abbreviations

ACSF: artificial cerebral spinal fluid
AWS: alcohol withdrawal syndrome
CIE: chronic intermittent ethanol
DIV: days *in vitro*
DMSO: dimethyl sulfoxide
E18: embryonic day 18
EAV: EAVSLKPT
EtOH: ethanol
GABA: γ-aminobutyric acid
GABA_A_Rs: GABA_A_ receptors
H89: H-89 dihydrochloride hydrate
HBSS: Hank’s balanced salt solution
HEPES: N-2-hydroxy-ethylpiperazine-N-2-ethanesulfonic acid
HRP: horseradish peroxidase
*I*_tonic_: tonic current
mIPSC: miniature inhibitory postsynaptic current
P/S: penicillin/streptomycin
PAGE: polyacrylamide gel electrophoresis
PBS: phosphate-buffered saline
PDBu: phorbol 12,13-dibutyrate
PDL: poly-D-lysine
PKA: protein kinase A
PKC: protein kinase C
PVDF: polyvinylidene fluoride
SDS: sodium dodecyl sulfate
TBS: Tris-buffered saline.

## References

[B1] Abrahao K. P. Salinas A. G. Lovinger D. M. (2017). Alcohol and the brain: neuronal molecular targets, synapses, and circuits. Neuron 96, 1223–1238. 10.1016/j.neuron.2017.10.032 29268093 PMC6566861

[B2] Abramian A. M. Comenencia-Ortiz E. Vithlani M. Tretter E. V. Sieghart W. Davies P. A.. (2010). Protein kinase C phosphorylation regulates membrane insertion of GABA_A_ receptor subtypes that mediate tonic inhibition. J. Biol. Chem. 285, 41795–41805. 10.1074/jbc.m110.149229 20940303 PMC3009907

[B3] Bohnsack J. P. Carlson S. L. Morrow A. L. (2016). Differential regulation of synaptic and extrasynaptic α4 GABA_A_ receptor populations by protein kinase A and protein kinase C in cultured cortical neurons. Neuropharmacology 105, 124–132. 10.1016/j.neuropharm.2016.01.009 26767953 PMC4873390

[B4] Bowers B. J. Owen E. H. Collins A. C. Abeliovich A. Tonegawa S. Wehner J. M. (1999). Decreased ethanol sensitivity and tolerance development in γ-protein kinase C null mutant mice is dependent on genetic background. Alcohol. Clin. Exp. Res. 23, 387–397. 10.1111/j.1530-0277.1999.tb04127.x 10195808

[B5] Bowers B. J. Wehner J. M. (2001). Ethanol consumption and behavioral impulsivity are increased in protein kinase Cγ null mutant mice. J. Neurosci. 21:RC180. 10.1523/jneurosci.21-21-j0004.2001 11606660 PMC6762808

[B6] Brickley S. G. Mody I. (2012). Extrasynaptic GABA_A_ receptors: their function in the CNS and implications for disease. Neuron 73, 23–34. 10.1016/j.neuron.2011.12.012 22243744 PMC3399243

[B7] Bright D. P. Smart T. G. (2013). Protein kinase C regulates tonic GABA_A_ receptor-mediated inhibition in the hippocampus and thalamus. Eur. J. Neurosci. 38, 3408–3423. 10.1111/ejn.12352 24102973 PMC4165308

[B8] Cagetti E. Liang J. Spigelman I. Olsen R. W. (2003). Withdrawal from chronic intermittent ethanol treatment changes subunit composition, reduces synaptic function, and decreases behavioral responses to positive allosteric modulators of GABA_A_ receptors. Mol. Pharmacol. 63, 53–64. 10.1124/mol.63.1.53 12488536

[B9] Caraiscos V. B. Elliott E. M. You-Ten K. E. Cheng V. Y. Belelli D. Newell J. G.. (2004). Tonic inhibition in mouse hippocampal CA1 pyramidal neurons is mediated by α5 subunit-containing γ-aminobutyric acid type A receptors. Proc. Natl. Acad. Sci. U S A 101, 3662–3667. 10.1073/pnas.0307231101 14993607 PMC373519

[B10] Carlson S. L. Bohnsack J. P. Morrow A. L. (2016a). Ethanol regulation of synaptic GABA_A_ α4 receptors is prevented by protein kinase A activation. J. Pharmacol. Exp. Ther. 357, 10–16. 10.1124/jpet.115.230417 26857960 PMC4809316

[B11] Carlson S. L. Bohnsack J. P. Patel V. Morrow A. L. (2016b). Regulation of extrasynaptic GABA_A_ α4 receptors by ethanol-induced protein kinase, A, but not protein kinase C activation in cultured rat cerebral cortical neurons. J. Pharmacol. Exp. Ther. 356, 148–156. 10.1124/jpet.115.228056 26483396 PMC4702069

[B12] Carlson S. L. O’Buckley T. K. Thomas R. Thiele T. E. Morrow A. L. (2014). Altered GABA_A_ receptor expression and seizure threshold following acute ethanol challenge in mice lacking the RIIβ subunit of PKA. Neurochem. Res. 39, 1079–1087. 10.1007/s11064-013-1167-0 24104609 PMC3981963

[B13] Chapell R. Bueno O. F. Alvarez-Hernandez X. Robinson L. C. Leidenheimer N. J. (1998). Activation of protein kinase C induces γ-aminobutyric acid type A receptor internalization in *Xenopus* oocytes. J. Biol. Chem. 273, 32595–32601. 10.1074/jbc.273.49.32595 9829997

[B14] Choi D. S. Wei W. Deitchman J. K. Kharazia V. N. Lesscher H. M. McMahon T.. (2008). Protein kinase Cδ regulates ethanol intoxication and enhancement of GABA-stimulated tonic current. J. Neurosci. 28, 11890–11899. 10.1523/JNEUROSCI.3156-08.2008 19005054 PMC2688726

[B16] Farrant M. Nusser Z. (2005). Variations on an inhibitory theme: phasic and tonic activation of GABA_A_ receptors. Nat. Rev. Neurosci. 6, 215–229. 10.1038/nrn1625 15738957

[B17] Glykys J. Mann E. O. Mody I. (2008). Which GABA_A_ receptor subunits are necessary for tonic inhibition in the hippocampus? J. Neurosci. 28, 1421–1426. 10.1523/JNEUROSCI.4751-07.2008 18256262 PMC6671570

[B18] Gonzalez C. Moss S. J. Olsen R. W. (2012). Ethanol promotes clathrin adaptor-mediated endocytosis via the intracellular domain of δ-containing GABA_A_ receptors. J. Neurosci. 32, 17874–17881. 10.1523/jneurosci.2535-12.2012 23223306 PMC3601804

[B19] Gordon A. S. Yao L. Wu Z. L. Coe I. R. Diamond I. (1997). Ethanol alters the subcellular localization of δ- and ε protein kinase C in NG108–15 cells. Mol. Pharmacol. 52, 554–559. 10.1124/mol.52.4.554 9380017

[B20] Hanchar H. J. Dodson P. D. Olsen R. W. Otis T. S. Wallner M. (2005). Alcohol-induced motor impairment caused by increased extrasynaptic GABA_A_ receptor activity. Nat. Neurosci. 8, 339–345. 10.1038/nn1398 15696164 PMC2854077

[B21] Harris R. A. McQuilkin S. J. Paylor R. Abeliovich A. Tonegawa S. Wehner J. M. (1995). Mutant mice lacking the γ isoform of protein kinase C show decreased behavioral actions of ethanol and altered function of γ-aminobutyrate type A receptors. Proc. Natl. Acad. Sci. U S A 92, 3658–3662. 10.1073/pnas.92.9.3658 7731960 PMC42020

[B22] Hodge C. W. Mehmert K. K. Kelley S. P. McMahon T. Haywood A. Olive M. F.. (1999). Supersensitivity to allosteric GABA_A_ receptor modulators and alcohol in mice lacking PKCε. Nat. Neurosci. 2, 997–1002. 10.1038/14795 10526339

[B23] Jacob T. C. Moss S. J. Jurd R. (2008). GABA_A_ receptor trafficking and its role in the dynamic modulation of neuronal inhibition. Nat. Rev. Neurosci. 9, 331–343. 10.1038/nrn2370 18382465 PMC2709246

[B24] Kittler J. T. Chen G. Honing S. Bogdanov Y. McAinsh K. Arancibia-Carcamo I. L.. (2005). Phospho-dependent binding of the clathrin AP2 adaptor complex to GABA_A_ receptors regulates the efficacy of inhibitory synaptic transmission. Proc. Natl. Acad. Sci. U S A 102, 14871–14876. 10.1073/pnas.0506653102 16192353 PMC1253579

[B25] Kittler J. T. Delmas P. Jovanovic J. N. Brown D. A. Smart T. G. Moss S. J. (2000). Constitutive endocytosis of GABA_A_ receptors by an association with the adaptin AP2 complex modulates inhibitory synaptic currents in hippocampal neurons. J. Neurosci. 20, 7972–7977. 10.1523/jneurosci.20-21-07972.2000 11050117 PMC6772725

[B26] Kumar S. Porcu P. Werner D. F. Matthews D. B. Diaz-Granados J. L. Helfand R. S.. (2009). The role of GABA_A_ receptors in the acute and chronic effects of ethanol: a decade of progress. Psychopharmacology 205, 529–564. 10.1007/s00213-009-1562-z 19455309 PMC2814770

[B27] Kumar S. Ren Q. Beckley J. H. O’Buckley T. K. Gigante E. D. Santerre J. L.. (2012). Ethanol activation of protein kinase A regulates GABA_A_ receptor subunit expression in the cerebral cortex and contributes to ethanol-induced hypnosis. Front. Neurosci. 6:44. 10.3389/fnins.2012.00044 22509146 PMC3321501

[B28] Kumar S. Suryanarayanan A. Boyd K. N. Comerford C. E. Lai M. A. Ren Q.. (2010). Ethanol reduces GABA_A_ α1 subunit receptor surface expression by a protein kinase Cγ-dependent mechanism in cultured cerebral cortical neurons. Mol. Pharmacol. 77, 793–803. 10.1124/mol.109.063016 20159950 PMC2872966

[B29] Laurie D. J. Wisden W. Seeburg P. H. (1992). The distribution of thirteen GABA_A_ receptor subunit mRNAs in the rat brain. III. Embryonic and postnatal development. J. Neurosci. 12, 4151–4172. 10.1523/jneurosci.12-11-04151.1992 1331359 PMC6576006

[B30] Liang J. Cagetti E. Olsen R. W. Spigelman I. (2004). Altered pharmacology of synaptic and extrasynaptic GABA_A_ receptors on CA1 hippocampal neurons is consistent with subunit changes in a model of alcohol withdrawal and dependence. J. Pharmacol. Exp. Ther. 310, 1234–1245. 10.1124/jpet.104.067983 15126642

[B31] Liang J. Suryanarayanan A. Abriam A. Snyder B. Olsen R. W. Spigelman I. (2007). Mechanisms of reversible GABA_A_ receptor plasticity after ethanol intoxication. J. Neurosci. 27, 12367–12377. 10.1523/JNEUROSCI.2786-07.2007 17989301 PMC6673253

[B32] Liang J. Zhang N. Cagetti E. Houser C. R. Olsen R. W. Spigelman I. (2006). Chronic intermittent ethanol-induced switch of ethanol actions from extrasynaptic to synaptic hippocampal GABA_A_ receptors. J. Neurosci. 26, 1749–1758. 10.1523/JNEUROSCI.4702-05.2006 16467523 PMC6793625

[B33] Mangan P. S. Sun C. Carpenter M. Goodkin H. P. Sieghart W. Kapur J. (2005). Cultured hippocampal pyramidal neurons express two kinds of GABA_A_ receptors. Mol. Pharmacol. 67, 775–788. 10.1124/mol.104.007385 15613639

[B34] Mehta A. K. Ticku M. K. (1999). An update on GABA_A_ receptors. Brain Res. Brain Res. Rev. 29, 196–217. 10.1016/s0165-0173(98)00052-6 10209232

[B35] Messing R. O. Petersen P. J. Henrich C. J. (1991). Chronic ethanol exposure increases levels of protein kinase C δ and ε and protein kinase C-mediated phosphorylation in cultured neural cells. J. Biol. Chem. 266, 23428–23432. 1744136

[B36] Mihalek R. M. Bowers B. J. Wehner J. M. Kralic J. E. VanDoren M. J. Morrow A. L.. (2001). GABA_A_-receptor δ subunit knockout mice have multiple defects in behavioral responses to ethanol. Alcohol. Clin. Exp. Res. 25, 1708–1718. 10.1111/j.1530-0277.2001.tb02179.x 11781502

[B37] Mody I. Pearce R. A. (2004). Diversity of inhibitory neurotransmission through GABA_A_ receptors. Trends Neurosci. 27, 569–575. 10.1016/j.tins.2004.07.002 15331240

[B38] Nakamura Y. Darnieder L. M. Deeb T. Z. Moss S. J. (2015). Regulation of GABA_A_Rs by phosphorylation. Adv. Pharmacol. 72, 97–146. 10.1016/bs.apha.2014.11.008 25600368 PMC5337123

[B39] Olive M. F. Mehmert K. K. Messing R. O. Hodge C. W. (2000). Reduced operant ethanol self-administration and *in vivo* mesolimbic dopamine responsiveness to ethanol in PKCε-deficient mice. The Eur. J. Neurosci. 12, 4131–4140. 10.1046/j.1460-9568.2000.00297.x 11069609

[B40] Olsen R. W. Hanchar H. J. Meera P. Wallner M. (2007). GABA_A_ receptor subtypes: the “one glass of wine” receptors. Alcohol 41, 201–209. 10.1016/j.alcohol.2007.04.006 17591543 PMC2852584

[B41] Olsen R. W. Liang J. (2017). Role of GABA_A_ receptors in alcohol use disorders suggested by chronic intermittent ethanol (CIE) rodent model. Mol. Brain 10:45. 10.1186/s13041-017-0325-8 28931433 PMC5605989

[B42] Olsen R. W. Sieghart W. (2008). International union of pharmacology. LXX. Subtypes of γ-aminobutyric Acid_A_ receptors: classification on the basis of subunit composition, pharmacology, and function. Update. Pharmacol. Rev. 60, 243–260. 10.1124/pr.108.00505 18790874 PMC2847512

[B43] Olsen R. W. Spigelman I. (2012). “GABA_A_ receptor plasticity in alcohol withdrawal,” in Jasper’s Basic Mechanisms of the Epilepsies, 4th Edn. eds Noebels J. L. Avoli M. Rogawski M. A. Olsen R. W. Delgado-Escueta A. V. (Bethesda, MD: Oxford University Press), 562–573.

[B44] Peng Z. Hauer B. Mihalek R. M. Homanics G. E. Sieghart W. Olsen R. W.. (2002). GABA_A_ receptor changes in δ subunit-deficient mice: altered expression of α4 and γ2 subunits in the forebrain. J. Comp. Neurol. 446, 179–197. 10.1002/cne.10210 11932935

[B46] Puia G. Vicini S. Seeburg P. H. Costa E. (1991). Influence of recombinant γ-aminobutyric acid-A receptor subunit composition on the action of allosteric modulators of γ-aminobutyric acid-gated Cl- currents. Mol. Pharmacol. 39, 691–696. 1646944

[B47] Qi Z. H. Song M. Wallace M. J. Wang D. Newton P. M. McMahon T.. (2007). Protein kinase Cε regulates γ-aminobutyrate type A receptor sensitivity to ethanol and benzodiazepines through phosphorylation of γ2 subunits. J. Biol. Chem. 282, 33052–33063. 10.1074/jbc.M707233200 17875639

[B48] Rudolph U. Crestani F. Möhler H. (2001). GABA_A_ receptor subtypes: dissecting their pharmacological functions. Trends Pharmacol. Sci. 22, 188–194. 10.1016/s0165-6147(00)01646-1 11282419

[B49] Semyanov A. Walker M. C. Kullmann D. M. Silver R. A. (2004). Tonically active GABA_A_ receptors: modulating gain and maintaining the tone. Trends Neurosci. 27, 262–269. 10.1016/j.tins.2004.03.005 15111008

[B50] Shen Y. Lindemeyer A. K. Spigelman I. Sieghart W. Olsen R. W. Liang J. (2011). Plasticity of GABA_A_ receptors after ethanol pre-exposure in cultured hippocampal neurons. Mol. Pharmacol. 79, 432–442. 10.1124/mol.110.068650 21163967 PMC3061361

[B51] Shen Y. Qin H. Chen J. Mou L. He Y. Yan Y.. (2016). Postnatal activation of TLR4 in astrocytes promotes excitatory synaptogenesis in hippocampal neurons. J. Cell Biol. 215, 719–734. 10.1083/jcb.201605046 27920126 PMC5147000

[B52] Sperk G. Schwarzer C. Tsunashima K. Fuchs K. Sieghart W. (1997). GABA_A_ receptor subunits in the rat hippocampus I: immunocytochemical distribution of 13 subunits. Neuroscience 80, 987–1000. 10.1016/s0306-4522(97)00146-2 9284055

[B53] Sundstrom-Poromaa I. Smith D. H. Gong Q. H. Sabado T. N. Li X. Light A.. (2002). Hormonally regulated α_4_β_2_δ GABA_A_ receptors are a target for alcohol. Nat. Neurosci. 5, 721–722. 10.1038/nn888 12118257 PMC2887346

[B54] Wallner M. Hanchar H. J. Olsen R. W. (2003). Ethanol enhances α_4_β_3_δ and α_6_β_3_δ γ-aminobutyric acid type A receptors at low concentrations known to affect humans. Proc. Natl. Acad. Sci. U S A 100, 15218–15223. 10.1073/pnas.2435171100 14625373 PMC299963

[B55] Wei W. Faria L. C. Mody I. (2004). Low ethanol concentrations selectively augment the tonic inhibition mediated by δ subunit-containing GABA_A_ receptors in hippocampal neurons. J. Neurosci. 24, 8379–8382. 10.1523/JNEUROSCI.2040-04.2004 15385620 PMC6729680

[B56] Wei W. Zhang N. Peng Z. Houser C. R. Mody I. (2003). Perisynaptic localization of δ subunit-containing GABA_A_ receptors and their activation by GABA spillover in the mouse dentate gyrus. J. Neurosci. 23, 10650–10661. 14627650 10.1523/JNEUROSCI.23-33-10650.2003 PMC6740905

[B57] Werner D. F. Kumar S. Criswell H. E. Suryanarayanan A. Fetzer J. A. Comerford C. E.. (2011). PKCγ is required for ethanol-induced increases in GABA_A_ receptor α4 subunit expression in cultured cerebral cortical neurons. J. Neurochem. 116, 554–563. 10.1111/j.1471-4159.2010.07140.x 21155805 PMC3033448

[B58] Werner D. F. Porcu P. Boyd K. N. O’Buckley T. K. Carter J. M. Kumar S.. (2016). Ethanol-induced GABA_A_ receptor α4 subunit plasticity involves phosphorylation and neuroactive steroids. Mol. Cell. Neurosci. 72, 1–8. 10.1016/j.mcn.2016.01.002 26805653 PMC4932829

[B59] Whiting P. J. Bonnert T. P. McKernan R. M. Farrar S. Le Bourdellès B. Heavens R. P.. (1999). Molecular and functional diversity of the expanding GABA-A receptor gene family. Ann. N Y Acad. Sci. 868, 645–653. 10.1111/j.1749-6632.1999.tb11341.x 10414349

